# Positive Association Between Ultrasonographic Fatty Liver Indicator and the Severity of Coronary Artery Disease

**DOI:** 10.3390/diagnostics15101208

**Published:** 2025-05-10

**Authors:** Tingqiu Wang, Zhigang Wang, Peng Luo

**Affiliations:** Department of Ultrasound, The Second Affiliated Hospital of Chongqing Medical University, Chongqing 400010, China; w857683014@163.com (T.W.); wzg62942443@163.com (Z.W.)

**Keywords:** coronary artery disease, metabolic dysfunction-associated fatty liver disease, SYNTAX score, ultrasonographic fatty liver indicator, ultrasonography

## Abstract

**Background**: This study investigates the link between metabolic dysfunction-associated fatty liver disease (MAFLD) and coronary artery disease (CAD) using the ultrasonographic fatty liver indicator (US-FLI) to assess liver steatosis. **Methods**: A total of 204 patients were included, with hepatic steatosis evaluated through ultrasound characteristics, diagnosing fatty liver when US-FLI was ≥2. CAD severity was determined using the SYNTAX score (SS), categorizing 100 CAD patients into mild (SS ≤ 22) and moderate-severe (MS) (SS ≥ 23) groups. The association between US-FLI and SS in patients with MAFLD was evaluated through the multivariate logistic regression model. A receiver operating characteristic curve was applied to determine the accuracy, sensitivity, and specificity of US-FLI in predicting SS. **Results**: In the multivariate logistic regression analysis, US-FLI was an independent predictor of the CAD group (OR = 1.194, 95% CI: 1.008–1.414, *p* = 0.040) and the MS group (OR = 1.262, 95% CI: 1.025–1.553, *p* = 0.028). In the receiver operating characteristic curve analysis, a US-FLI value of 2 was found to be the optimal threshold point for diagnosing MS CAD patients (AUC = 0.620, 95% CI: 0.509–0.713, *p* = 0.039), with a sensitivity of 65.22% and a specificity of 55.56%. The diagnostic performance of MS CAD patients significantly improved when US-FLI was combined with type 2 diabetes mellitus (T2DM) (AUC = 0.732, 95% CI: 0.632–0.832, *p* < 0.001), with a sensitivity of 65.22% and specificity of 77.78%. **Conclusions**: US-FLI was independently and positively associated with CAD severity. US-FLI combined with T2DM had better diagnostic performance in patients with MS CAD.

## 1. Introduction

Nonalcoholic fatty liver disease (NAFLD) refers to the fatty degeneration of liver cells exceeding 5% in the absence of other liver-damaging factors. NAFLD is the most prevalent chronic liver disease globally and the leading cause of liver-related morbidity and mortality, accounting for around 30% of cases [1]. NAFLD-related cirrhosis and hepatocellular cancer are the primary reasons for liver transplantation [2]. NAFLD is a multisystem disease that increases the risk of extrahepatic organ diseases [3]. A meta-analysis showed that NAFLD was independently related to the occurrence of coronary artery disease (CAD) (relative risk = 1.21, 95% CI: 1.07–1.38) [4]. Coronary heart disease is the leading cause of mortality in China, and a notable epidemiological aspect of the country is the sharp increase in the atherosclerotic cardiovascular disease (CVD) burden [5]. Recent research has revealed a strong association between metabolism and the aetiology and symptoms of fatty liver. An international expert consensus recommends substituting the term “metabolic dysfunction-associated fatty liver disease (MAFLD)” for “NAFLD” [6]. Whether patients have MAFLD or NAFLD, CVD is the leading cause of death for them [7,8].

Although a liver biopsy is the most reliable method for identifying chronic liver disease, it is not appropriate for large-scale population screening because of its invasiveness, sampling variability, and uncommon complications. Owing to changes in contemporary living environments and lifestyles and advancements in diagnostic and treatment techniques, fatty liver disease (FLD) has been identified as a global pandemic affecting hundreds of millions of people. Hepatic fat degeneration is a prerequisite for diagnosing NAFLD/MAFLD. The development of non-invasive liver disease assessment tools, including imaging methods and laboratory indicators, has improved FLD diagnosis and prognosis [9]. A study investigating the relationship between hepatic steatosis and CAD showed that patients with CAD and hepatic steatosis had a worse prognosis than those without hepatic steatosis [10]. In this study, hepatic steatosis was evaluated using CT. However, the disadvantages of CT, such as radioactivity and high price, limit its promotion among the general public. Ultrasound is an inexpensive, quick, and safe diagnostic technique with 89% sensitivity and 93% specificity for identifying FLD [11]. Building on liver biopsy as the gold standard in their research, Ballestri et al. developed the ultrasonographic fatty liver indicator (US-FLI), a semiquantitative index, to assess the degree of liver steatosis [12], wherein a higher US-FLI value indicates a higher degree of liver steatosis. With US-FLI ≥ 2, the sensitivity and specificity of detecting mild fatty liver were 90.1% and 90%, respectively. One study found that US-FLI as a continuous variable could predict the occurrence of NAFLD in children [13]. The SYNTAX score (SS) is a risk model that stratifies CAD risk based on the invasive coronary angiography (ICA) according to the anatomical morphological characteristics of lesions, such as the number and location of diseased vessels, involved coronary arteries, calcification, coronary artery course, degree of occlusion, and thrombosis, to determine the best revascularization strategy and predict prognosis [14]. The higher the SS, the more complicated the disease, indicating considerable treatment challenges and a worse prognosis.

Since the term “MAFLD” is relatively new, most clinical research is focused on the non-invasive diagnosis of NAFLD, while there are relatively few non-invasive diagnostic studies based on MAFLD. This study used US-FLI to determine the level of hepatic fatty degeneration in patients with MAFLD and examined its correlation with SS.

## 2. Materials and Methods

### 2.1. Study Population

**Figure 1:**
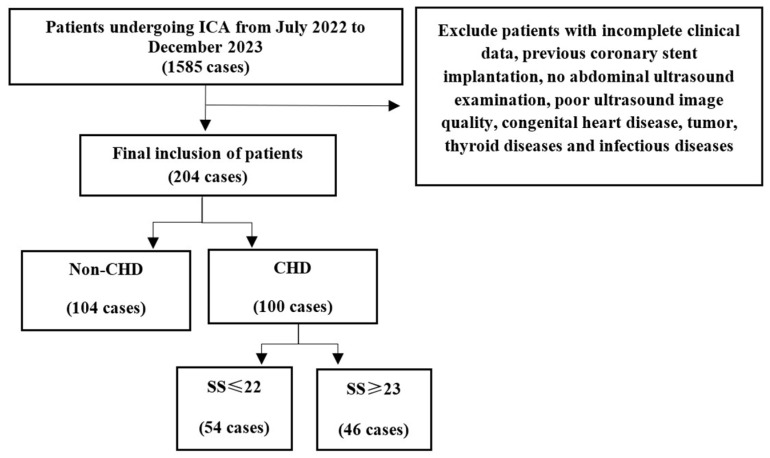
Sample inclusion flow chart.

This retrospective study received approval from our hospital’s Ethics Committee, which also granted a waiver for the patients’ informed consent forms. This cross-sectional study was conducted at the Department of Cardiology of the Second Affiliated Hospital of Chongqing Medical University. The inclusion criteria were patients aged 18 years and older who visited our hospital for chest pain, chest tightness, or other reasons and underwent ICA from July 2022 to December 2023. This study excluded patients with incomplete clinical data, previous coronary stent implantation, no abdominal ultrasound examination, poor ultrasound image quality, congenital heart disease, tumor, thyroid diseases, and infectious diseases. In all, 204 patients were involved in this investigation. The SS is an angiographic tool for diagnosing CAD and grading CAD complexity. Experienced cardiologists blinded to the patient’s other information performed the ICA within one week of the echocardiogram. The severity of CAD was assessed utilizing the SYNTAX scoring tool (https://syntaxscore.org/, accessed on 1 December 2023). Every coronary artery lesion with luminal stenosis of at least 50% and a vessel diameter of at least 1.5 mm was scored. The specific scoring principles are as follows: ① severe CAD: SS ≥ 33; ② moderate CAD: 23 ≤ SS ≤ 32; ③ mild CAD: SS ≤ 22. The patients were further divided into the mild group (SS ≤ 22) and moderate-severe (MS) group (SS ≥ 23). Figure 1 illustrates this procedure.

### 2.2. Definition of MAFLD

MAFLD is defined as hepatic steatosis evidence-based on histology, blood biomarkers, or imaging, combined with one of the following three criteria: T2DM, overweight/obesity (body mass index (BMI) ≥ 23 kg/m^2^), or the presence of two or more metabolic abnormalities. Metabolic abnormalities include the following: (1) waist circumference ≥ 102 cm in males or waist circumference ≥ 88 cm in females, (2) ≥blood pressure is 130/85 mmHg or under specific antihypertensive medication, (3) triglyceride (TG) in plasma ≥ 1.70 mmol/L or under specific anti-cholesterol medication, (4) HDL-C (high-density lipoprotein cholesterol) < 1.0 mmol/L for males and <1.3 mmol/L for females or specific drug treatment, (5) prediabetes, defined as 2 h post-load glucose levels between 7.8 and 11.0 mmol, or fasting glucose levels between 5.6 and 6.9 mmol/L, or HbA1c levels between 5.7% and 6.4%, (6) evaluation of insulin resistance (IR) score ≥ 2.5 using the homeostasis model, and (7) hsCRP > 2 mg/L.

**Figure 2:**
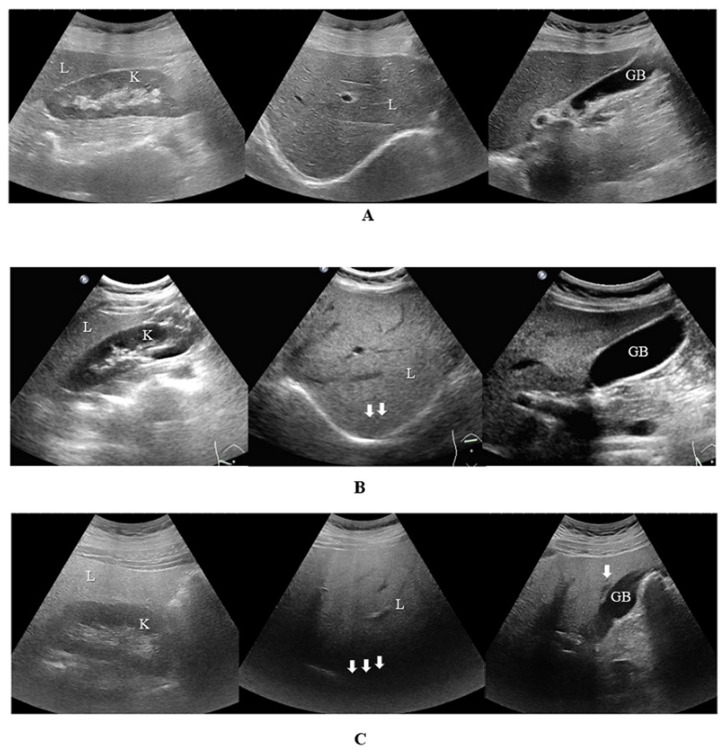
Calculation of US-FLI. Figures ABC show ultrasound images of liver sections in three patients. (**A**) Comparison of liver and kidney (score 0), US-FLI = 0. (**B**) Comparison of liver and kidney (score 3), posterior echo attenuation (score 1) (two arrows), blurring of the vessels (score 1), US-FLI = 5. (**C**) Comparison of liver and kidney (score 3), posterior echo attenuation (score 1), blurring of the vessels (score 1), unclear display of gallbladder wall (score 1), difficulty in displaying diaphragm (score 1) (three arrows), difficult visualization of the areas of focal sparing (score 1) (one arrow), US-FLI = 8. Abbreviations: L, liver; K, kidney; GB, gallbladder.

The degree of hepatic steatosis was assessed using US-FLI. The US-FLI value increases with the degree of hepatic steatosis. The patient was asked to fast for 8–12 h before the abdominal ultrasound examination and maintain a horizontal position during the examination. Two diagnostic medical sonographers with more than two years of work experience used a 3.5 MHz convex array probe to scan each liver section. Patients with clear images were included in the study. The range of US-FLI is 0–8. The scoring rules were as follows: ① liver and kidney contrast imaging for fatty liver diagnosis; ② a posteriorly attenuated ultrasonic beam; ③ blurring of vessels; ④ difficulty in visualizing gallbladder wall; ⑤ problematic diaphragm visualization; and ⑥ low-fat area retention [12]. Fatty liver was diagnosed when US-FLI ≥ 2. Figure 2 shows the US-FLI calculation process for patients in our hospital.

### 2.3. CVD Risk Factor Indicators

Blood samples were drawn for laboratory tests upon admission before the patients started taking any medication. Laboratory tests were performed immediately after sampling at the central laboratory of the hospital. The patient’s complete blood count, which included red blood cell (RBC), neutrophil (NEUT), monocyte (MONO), lymphocyte (LYM), and platelet (PLT) counts, was recorded at admission. Blood lipid-related indicators included lipoprotein (a) (Lp(a)), low-density lipoprotein cholesterol (LDL-C), HDL-C, total cholesterol (T-Cho), apolipoprotein A1 (ApoA1), non-esterified fatty acid (NEFA), and TG. In addition, heart-related indicators exist. After resting for 10 min, the resting heart rate (RHR) was measured using a 24 h dynamic electrocardiogram, and the patient’s ejection fraction (EF) was obtained from the echocardiography report. Liver function indicators included alanine aminotransferase (ALT), alkaline phosphatase (ALP), aspartate aminotransferase (AST), glutamyl transaminase (GGT), and globulin (GLB). The patient’s family history of CAD, type of work, diet, and physical activity over 150 min a week were obtained from the patient’s medical history. Dietary habits were assessed according to the Mediterranean diet assessment tool [15]. T2DM was diagnosed if one of the following four requirements was met: (1) taking glucose-lowering drugs; (2) HbA1c ≥ 6.5% (48 mmol/mol); (3) fasting plasma glucose ≥ 7.0 mmol/L (126 mg/dL); (4) 2 h plasma glucose (2hPG) ≥ 11.1 mmol/L (200 mg/dL). When one of the two following criteria was met, hypertension (HBP) was diagnosed: (1) antihypertensive medication use; or (2) blood pressure ≥ 130/85 mmHg.

### 2.4. Statistical Analysis

Statistical analysis was performed using IBM SPSS statistical software (version 26) and R language (version 4.2.2). Continuous variables are expressed as mean ± standard deviation or median (interquartile range), and categorical variables are expressed as counts (percentages). The analysis of interobserver agreement in the US-FLI employed the intraclass correlation coefficient. The Kolmogorov–Smirnov test was used to test the normality of continuous variables. A T-test was used for comparison of the variables that met the normality test criteria. The Mann–Whitney U test was used for non-normally distributed data. The chi-squared test or Fisher’s exact test was used to analyze categorical variables. Using the SS binary classification as the outcome variable, univariate and multivariate regression analyses were used to screen for variables associated with the severity of coronary artery lesions. Variables with *p* < 0.2 in univariate regression analysis were entered into multivariate regression analysis. Multivariate linear regression analysis was used to assess the collinearity between variables, and odds ratios (ORs) and their 95% confidence intervals (CIs) were used to quantify the strength of the association between the two variables. Receiver operating characteristic (ROC) curves were used to determine the accuracy, sensitivity, and specificity of the study variables for predicting SS. A logistic regression model was established based on screened variables. A two-tailed *p* < 0.05 was defined as statistical significance.

## 3. Results

### 3.1. Baseline Characteristics

This study comprised 204 eligible patients in total, with an average age of 67 years, of which 53.9% were male, and 50.0%, 30.9%, and 61.8% had MAFLD, T2DM, and HBP, respectively. Among these participants, 100 were diagnosed with CAD using ICA. The intraclass correlation coefficient for the US-FLI was 0.948 ((0.922–0.964), *p* < 0.001), indicating a high level of inter-group consistency (Table S1). Therefore, we utilized the average score from both groups to represent the US-FLI. As shown in Table 1, patients in the CAD group were significantly older than those in the non-CAD group, had a larger number of smokers and were male, and had a larger proportion of patients with T2DM or HBP than those in the non-CAD group. ALT, AST, GGT, NEUT, MONO, and hsCRP were significantly higher, and HDL-C and ApoA1 levels were significantly lower in the CAD group than in the non-CAD group. US-FLI in the CAD group was higher than that in the non-CAD group (2.00 (0.00–4.00) vs. 0.00 (0.00–3.00), *p* = 0.038).

The SYNTAX scoring tool was used to further divide CAD patients into a mild group (SS ≤ 22) and an MS group (SS ≥ 23), with a total of 46 patients in the MS group. Compared with the mild group, patients in the MS group were older and had a higher prevalence of T2DM; however, there were no differences in sex, smoking, or drinking. The US-FLI in the MS group was higher than that in the mild group (3.00 (0.00–4.00) vs. 0.00 (0.00–3.00), *p* = 0.029). Regarding cardiac function, EF was lower in the MS group than in the mild group (67.00 (60.75–70.00) vs. 70.00 (66.25–74.75), *p* = 0.020) (Table 2).

### 3.2. Multivariate Logistic Regression Analysis

To further explore the independent predictors of the CAD group and the MS group, we included the variables with *p* < 0.2 in the univariate regression analysis into the multivariate regression model based on the traditional and non-traditional risk factors affecting SS. After adjusting for US-FLI, sex, age, smoking, EF, T2DM, HBP, AST, HDL-C, ApoA1, cholinesterase (ChE), NEUT, and MONO, we found that US-FLI (OR = 1.194, 95% CI: 1.008–1.414, *p* = 0.040) was positively correlated with the risk of CAD. Age (OR = 1.054, 95% CI: 1.014–1.095, *p* = 0.008), smoking (OR = 2.581, 95% CI: 1.016–6.552, *p* = 0.046), and HBP (OR = 2.974, 95% CI: 1.463–6.046, *p* = 0.003) were independent risk factors for patients in the CAD group (Table 3). Collinearity diagnosis revealed no multicollinearity among the variables (Table 4).

After adjusting for US-FLI, sex, EF, T2DM, HBP, TBIL, ApoA1, and physical activity, it was discovered that US-FLI and the risk of CAD were positively associated (OR = 1.262, 95% CI: 1.025–1.553, *p* = 0.028), and T2DM (OR = 3.337, 95% CI: 1.226–9.085, *p* = 0.018) was identified as an independent risk factor for patients in the intermediate-risk group (Table 5). Collinearity diagnosis revealed no multicollinearity among the variables (Table 6).

### 3.3. The Diagnostic Value of US-FLI for MS CAD

**Figure 3:**
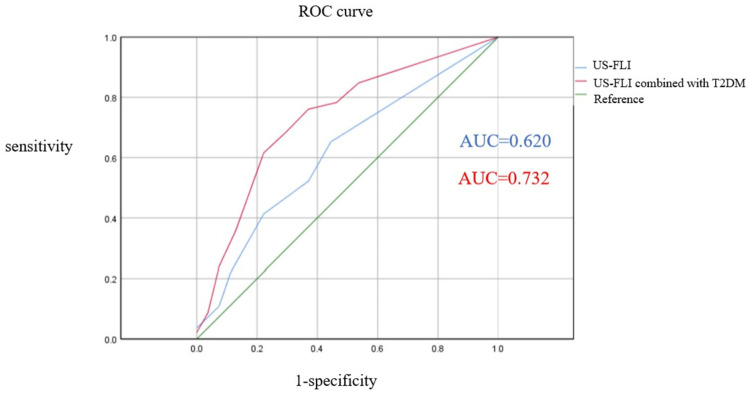
Using ROC curves to assess US-FLI’s diagnostic performance for coronary artery disease severity.

The ROC curve was used to analyze the detection efficiency of SS-based US-FLI for MS CAD. The US-FLI value of 2 was considered the optimal threshold point for diagnosing patients with MS CAD (AUC = 0.620, 95% CI: 0.509–0.713, *p* = 0.039), with a sensitivity of 65.22% and a specificity of 55.56%; when US-FLI was combined with T2DM, the diagnostic performance of MS CAD significantly improved (AUC = 0.732, 95% CI: 0.632–0.832, *p* < 0.001), with a sensitivity of 65.22% and a specificity of 77.78% (Figure 3).

## 4. Discussion

Our results showed that the new semiquantitative ultrasound liver steatosis index, US-FLI, was an independent predictor for CAD and positively associated with the severity of CAD after adjusting for confounders. For each increase of 1 unit of US-FLI, the probability of MS CAD increased by 1.262 times. In addition, we demonstrated that T2DM was an independent risk factor for MS CAD, and the risk of MS CAD in T2DM patients increased by 3.337 times compared with those without T2DM. US-FLI combined with T2DM showed better predictive performance in the prediction model of MS CAD risk than US-FLI alone. Consistent with prior research, our results indicated that male and patients with lower values of EF and TBIL, higher values of ApoA1, and less than 150 min of physical activity per week were more likely to have MS CAD, even though these were not statistically significant. To our knowledge, few studies have examined the correlation between steatosis and the severity of CAD in MAFLD using US-FLI.

As a multisystem disease, NAFLD/MAFLD is characterized by an increased risk of extrahepatic events, the most significant of which are cancer and CVD [16]. A large cohort study involving 5671 subjects found that patients with hepatic steatosis had an increased risk of early atherosclerosis [17]. A meta-analysis found the risk of CVD in patients with NAFLD increased by 57–69% after correcting for other common risk factors [18]. Even without T2DM or hypertension, patients with NAFLD showed a bias towards developing CAD in the next 10 years, revealing that NAFLD may pose an independent risk factor for CVD or that there are other undetermined risk factors. In addition, Toh et al., in a meta-analysis of 38 included articles, reported that the prevalence of CAD in patients with MS hepatic steatosis was 37.5%, which was significantly higher than that in patients with mild hepatic steatosis [7]. These findings coincide with those of this study. Metabolic syndrome (MetS) is a general term used to describe multiple CVD-related risk factors, including IR, atherosclerotic dyslipidaemia, central obesity, and HBP. A study using systems biology models showed a large overlap in the disease mechanisms of NAFLD and MetS [19]. Some studies have pointed out that NAFLD is a liver manifestation of MetS, and the term “MAFLD” was based on this concept. Although many studies have shown that MAFLD/NAFLD, as a multisystem disease, is strongly associated with CVD, the fundamental mechanism is still not fully elucidated. However, the following possibilities still exist.

Given the strong association between MetS and NAFLD/MAFLD, as well as their link to CVD, it is crucial to explore how shared risk factors contribute to these interactions. Among these, obesity stands out as a central factor influencing the development and progression of both conditions. As lifestyle changes, obesity has become a global epidemic and has shown a continuous upward trend over the past 50 years. According to estimates by the World Health Organization, 1.9 billion adults worldwide are overweight or obese, which brings with it an increased social burden and disease risks such as CVD, T2DM, and fatty liver [20]. Obesity is the abnormal accumulation of fat that can impair health, leading to metabolic changes in white adipose tissue and the induction of inflammation through adipokines, which promotes the progression of many chronic metabolic diseases. Adipokines such as adiponectin, leptin, and omentin are bioactive molecules secreted by adipose tissue. They influence MAFLD and CAD through mechanisms such as inflammation, IR, oxidative stress, and lipid metabolism, potentially leading to mutual influence between the two conditions. There are several methods for assessing obesity, including visceral fat accumulation and anthropometric indicators. Although BMI, as a measurement indicator of peripheral obesity, has been confirmed to be associated with CAD, some studies have reported that it is not the most appropriate indicator for predicting the severity of CAD, which may be related to the close correlation between central obesity and CAD [21,22,23]. This study explored the association of FLD, which is closely related to central obesity, with CAD. Although unclear, possible mechanisms by which obesity causes coronary atherosclerosis include the fact that the liver is exposed to elevated levels of NEFA due to the tendency of abdominal fat cells to excrete NEFA straight into the portal vein [24]. Increased NEFA levels lead to atherogenic lipid synthesis in the liver. Additionally, low-grade inflammation in the liver and fat caused by NEFA may lead to cardiovascular events. Systemic inflammation is associated with the onset and progression of atherosclerosis, while persistent chronic low-grade inflammation also indicates the risk of complications arising from atherosclerosis [25]. Even at the initial stages of lipid accumulation in the arterial wall, inflammatory cells such as leukocytes begin to localize and aggregate at the lesion site by binding to adhesion molecules expressed by endothelial cells of the artery [26]. The CAD is fundamentally a chronic immune-inflammatory fibroproliferative disorder induced by lipids [27]. In a large cohort study from the multi-ethnic study of atherosclerosis, the NAFLD group exhibited higher serum levels of inflammatory markers [28]. NAFLD induces systemic inflammation through complex interactions between the gut microbiota, liver, and adipose tissue. The pro-inflammatory cytokines released in this process may contribute to plaque formation and endothelial dysfunction, ultimately leading to CAD [29].

Studies have shown that NAFLD is one of the groups with the highest risk of developing T2DM [30]. The American Diabetes Association found that T2DM is an independent risk factor for NAFLD [31]. Brar et al. showed that a reduction in fatty liver degeneration could prevent diabetes mellitus [32]. High blood sugar levels, IR, and impaired pancreatic islet cell function are characteristics of T2DM. Although the mechanism remains unclear, it seems that IR is a key factor and has a bidirectional relationship with NAFLD. A study by FU C-P et al. showed that the blood glucose level 2 h after an oral glucose tolerance test was positively correlated with SS in patients with angina pectoris, which is consistent with the conclusion of our study [33]. Our study further combined US-FLI with T2DM and found that the combined index was more valuable for diagnosing the severity of CAD. IR is characterized by an abnormal cellular response to insulin, manifested as elevated NEFA, impaired blood glucose regulation, and hyperinsulinemia, indicative of metabolic dysfunction. It contributes to the development of NAFLD by altering glucose, lipid, and protein metabolism. Additionally, inflammation and visceral fat accumulation may further exacerbate IR. The study by Song J et al. reported that IR increases the risk of more severe CAD in patients with T2DM [34]. The connection between IR and coronary endothelial dysfunction is closely linked to inflammation and obesity, which intersect through various metabolic pathways, ultimately leading to atherosclerosis [35]. However, the precise mechanisms involved are still under investigation.

In a previous study, we found that the causes of MAFLD/NAFLD and CVD are intertwined and interrelated through metabolism-related mechanisms, thereby promoting their development. These findings highlight the complexity of chronic diseases. Fatty liver not only affects liver metabolism and causes liver damage but is also directly or indirectly linked with coronary atherosclerosis, ultimately damaging heart function. Conversely, the presence of coronary atherosclerosis also implicates the risk of MAFLD, thus confirming the hypothesis of a liver–heart axis. Any of these circumstances may lead to difficulties in disease diagnosis and treatment. Currently, the annual rates of morbidity and mortality for NAFLD/MAFLD and CVD are increasing. What we see now is just the tip of the iceberg; what lies beneath the surface is closer to the truth. However, early detection of the disease is equally important for identifying its pathophysiological mechanisms. Therefore, our study used fast, convenient, and harmless ultrasound examinations to assess liver fatty degeneration through new indicators to explore their relationship with the severity of CAD and to find a simple method to screen MS groups.

However, our study had certain restrictions. This was a small cross-sectional study, and we did not assess the prognostic value of US-FLI. Although ultrasound indicators have good diagnostic efficacy and can accurately assess the degree of fatty liver degeneration, a liver biopsy remains the most accurate method. As a result, the diagnostic sensitivity of US-FLI for MS CAD may be compromised. We were unable to completely rule out the influence of potential confounders, even after adjusting for a number of pertinent confounding variables, potentially affecting the study’s validity. Owing to data limitations, our study did not evaluate a range of inflammatory markers, including interleukin-6, a detailed medication history, and nutritional status, which may have implications for the observed associations. Since this study used manual measurements to evaluate fatty liver and coronary heart disease, this may also explain why the ultrasound fatty liver index was not sensitive enough to diagnose MS CAD. The results of this study are likely to be regionally limited in terms of population generalization. The evolving terminology and diagnostic criteria differences between metabolic dysfunction-associated steatotic liver disease and MAFLD also present challenges, indicating that future research could benefit from incorporating metabolic dysfunction-associated steatotic liver disease criteria to better understand its implications [36]. Therefore, further large-scale prospective studies are warranted.

## 5. Conclusions

US-FLI is an easily accessible, non-invasive ultrasound score for liver steatosis. We found that it is a promising noninvasive indicator for predicting the severity of CAD lesions. US-FLI is positively correlated with the severity of CAD, based on the SS assessment. Compared with US-FLI alone, US-FLI combined with T2DM has better diagnostic efficacy in patients with MS CAD. Specifically, among individuals with T2DM, a higher US-FLI is associated with more severe CAD. In clinical practice, US-FLI could be utilized as a screening tool to identify patients at higher risk of severe CAD, particularly among those with T2DM. Its non-invasive nature makes it an attractive option for regular monitoring and early intervention, potentially guiding treatment decisions and improving patient outcomes. Future studies are needed to further validate US-FLI’s utility in diverse populations and integrate it into existing cardiovascular risk assessment models.

## Figures and Tables

**Table 1:** Comparison of baseline characteristics between CAD group and non-CAD group.

	**Overall (*n* = 204)**	**Non-CAD (*n* = 104)**	**CAD (*n* = 100)**	***p***
Age, year	67.00 (59.00–72.00)	65.00 (58.00–69.00)	69.00 (63.00–74.00)	0.001 *
Male, (%)	110 (53.9)	47 (45.2)	63 (63.0)	0.016 *
Smoking, (%)	60 (29.4)	22 (21.2)	38 (38.0)	0.013 *
Drinking, (%)	22 (10.8)	10 (9.6)	12 (12.0)	0.747
HBP, (%)	126 (61.8)	52 (50.0)	74 (74.0)	0.001 *
T2DM, (%)	63 (30.9)	24 (23.1)	39 (39.0)	0.021 *
Family history, (%)	9 (4.4)	8 (5.1)	1 (2.2)	0.666
Job, (%)				0.930
Unemployed	164 (80.4)	83 (79.8)	81 (81.0)	
Employed	20 (9.8)	11 (10.6)	9 (9.0)	
Self-employed	20 (9.8)	10 (9.6)	10 (10.0)	
Mediterranean diet, (%)	27 (13.2)	17 (16.3)	10 (10.0)	0.258
Physical activity, (%)	110 (53.9)	57 (54.8)	53 (53.0)	0.906
MAFLD, (%)	102(50.0)	48 (46.2)	54 (54.0)	0.327
US-FLI	1.00 (0.00–3.00)	0.00 (0.00–3.00)	2.00 (0.00–4.00)	0.038 *
BMI, kg/m^2^	24.00 (22.02–26.38)	23.96 (21.92–26.51)	24.02 (22.12–26.31)	0.713
EF, %	69.00 (66.00–73.00)	70.00 (67.00–73.00)	69.00 (64.00–73.00)	0.094
RHR, bpm	75.00 (68.00–83.00)	75.00 (68.00–82.00)	76.00 (68.00–84.00)	0.809
ALP, U/L	72.50 (62.00–86.00)	70.00 (61.00–86.25)	75.50 (66.75–85.25)	0.103
ALT, U/L	22.00 (15.00–27.00)	19.00 (14.00–27.00)	25.50 (16.00–27.75)	0.008 *
AST, U/L	22.00 (18.00–28.00)	21.00 (18.00–26.00)	23.00 (18.75–34.25)	0.006 *
TBIL, μmol/L	11.80 (9.00–15.20)	12.00 (9.60–15.25)	11.25 (8.80–15.05)	0.370
GGT, U/L	25.00 (19.00–41.25)	22.00 (17.75–32.25)	30.00 (23.00–45.00)	<0.001 *
GLB, g/L	28.10 (25.50–31.92)	27.95(25.28–30.92)	28.85(25.80–32.42)	0.084
ApoA1, g/L	1.35 ± 0.27	1.40 ± 0.27	1.29 ± 0.26	0.003 *
HDL-C, mmol/L	1.21 (1.04–1.40)	1.25 (1.12–1.47)	1.14 (0.99–1.33)	0.003 *
LDL-C, mmol/L	2.65 (2.00–3.22)	2.79 (1.97–3.29)	2.54 (2.02–3.07)	0.263
Lp(a), mg/L	159.70 (85.10–324.68)	137.10 (73.33–351.50)	186.70 (96.82–299.55)	0.090
NEFA, mmol/L	0.38 (0.23–0.56)	0.35 (0.23–0.51)	0.41 (0.22–0.58)	0.396
T-Cho, mmol/L	0.56 ± 1.21	4.50 ± 1.21	4.40 ± 1.20	0.550
TG, mmol/L	1.23 (0.91–1.88)	1.17 (0.85–1.87)	1.29 (0.98–1.88)	0.284
HGB, g/L	135.58 ± 16.46	136.71 ± 13.91	134.41 ± 18.76	0.319
ChE, KU/L	8.47 ± 1.94	8.71 ± 1.90	8.23 ± 1.96	0.072
LYM, 10^9^/L	1.52 (1.22–1.88)	1.49 (1.19–1.79)	1.54 (1.22–1.96)	0.528
MONO, 10^9^/L	0.43 (0.34–0.52)	0.40 (0.33–0.47)	0.46 (0.35–0.56)	0.002 *
NEUT, 10^9^/L	3.70 (3.13–4.79)	3.57 (3.00–4.44)	3.78 (3.24–5.22)	0.037 *
RBC, 10^12^/L	4.50 ± 0.59	4.55 ± 0.52	4.44 ± 0.64	0.171
HsCRP, mg/L	1.69 [0.75, 3.87]	1.33 [0.55, 2.50]	2.26 [1.08, 4.86]	<0.001 *
PLT, 10^9^/L	194.00 (162.50–223.75)	194.50 (163.00–225.00)	194.00 (157.00–217.50)	0.821

Abbreviations: CAD, coronary artery disease; HBP, hypertension; T2DM, type 2 diabetes; MAFLD, metabolic dysfunction-associated fatty liver disease; US-FLI, ultrasonographic fatty liver indicator; BMI, body mass index; EF, ejection fraction; RHR, resting heart rate; ALP, alkaline phosphatase; ALT, alanine aminotransferase; AST, aspartate aminotransferase; TBIL, total bilirubin; GGT, glutamyltransaminase; GLB, globulin; ApoA1, apolipoprotein A1; HDL-C, high-density lipoprotein cholesterol; LDL-C, low-density lipoprotein cholesterol; Lp(a), lipoprotein (a); NEFA, non-esterified fatty acid; T-Cho, total cholesterol; TG, triglyceride; HGB, hemoglobin; ChE, cholinesterase; LYM, lymphocytes; MONO, monocytes; NEUT, neutrophils; RBC, red blood cells; PLT, platelets. *: *p* < 0.05.

**Table 2:** Baseline characteristics of participants based on SS.

	**Overall (*n* = 100)**	**SS ≤ 22 (*n* = 54)**	**SS ≥ 23 (*n* = 46)**	***p***
Age, year	67.85 ± 10.22	66.00 (58.00–70.00)	69.00 (63.00–74.00)	0.004 *
Male, (%)	63 (63.0)	30 (55.6)	33 (71.7)	0.144
Smoking, (%)	38 (38.0)	19 (35.2)	19 (41.3)	0.673
Drinking, (%)	12 (12.0)	8 (14.8)	4 (8.7)	0.529
HBP, (%)	74 (74.0)	44 (81.5)	30 (65.2)	0.105
T2DM, (%)	39 (39.0)	12 (22.2)	27 (58.7)	<0.001 *
Family history, (%)	3 (3.0)	2 (3.7)	1 (2.2)	0.999
Job, (%)				0.503
Unemployed	81 (81.0)	46 (85.2)	35 (76.1)	
Employed	9 (9.0)	4 (7.4)	5 (10.9)	
Self-employed	10 (10.0)	4 (7.4)	6 (13.0)	
Mediterranean diet, (%)	10 (10.0)	7 (13.0)	3 (6.5)	0.462
Physical activity, (%)	53 (53.0)	33 (61.1)	20 (43.5)	0.119
MAFLD, (%)	54 (54.0)	24 (44.4)	30 (65.2)	0.061
US-FLI	2.00 (0.00–4.00)	0.00 (0.00–3.00)	3.00 (0.00–4.00)	0.029 *
BMI, kg/m^2^	24.50 ± 3.54	24.18 ± 2.96	24.87 ± 4.11	0.334
EF, (%)	69.00 (64.00–73.00)	70.00 (66.25–74.75)	67.00 (60.75–70.00)	0.020 *
RHR, bpm	76.49 ± 12.38	75.30 ± 10.72	77.89 ± 14.08	0.299
ALP, U/L	75.50 (66.75–85.25)	72.00 (66.25–83.00)	79.00 (67.00–89.00)	0.187
ALT, U/L	25.50 (16.00–27.75)	26.50 (16.25–27.00)	22.50 (16.00–30.75)	0.554
AST, U/L	23.00 (18.75–34.25)	22.00 (19.00–33.75)	23.50 (18.00–35.75)	0.601
TBIL, μmol/L	11.25 (8.80–15.05)	11.45 (8.65–16.33)	11.05 (9.00–14.22)	0.459
GGT, U/L	30.00 (23.00–45.00)	28.00 (22.00–42.75)	31.50 (25.00–54.25)	0.159
GLB, g/L	29.48 ± 5.02	29.06 ± 4.43	29.96 ± 5.64	0.373
ApoA1, g/L	1.29 ± 0.26	1.32 ± 0.27	1.25 ± 0.25	0.141
HDL-C, mmol/L	1.18 ± 0.28	1.21 ± 0.30	1.14 ± 0.25	0.212
LDL-C, mmol/L	2.59 ± 0.82	2.49 ± 0.69	2.70 ± 0.94	0.195
Lp(a), mg/L	186.70 (96.82–299.55)	195.55 (88.05–318.82)	182.50 (114.08–294.08)	0.740
NEFA, mmol/L	0.41 (0.22–0.58)	0.38 (0.20–0.58)	0.42 (0.26–0.63)	0.589
T-Cho, mmol/L	4.40 ± 1.20	4.31 ± 1.11	4.50 ± 1.31	0.444
TG, mmol/L	1.29 (0.98–1.88)	1.23 (0.89–1.61)	1.35 (1.07–2.18)	0.096
HGB, g/L	134.41 ± 18.76	136.31 ± 18.11	132.17 ± 19.45	0.273
ChE, KU/L	8.23 ± 1.96	8.10 ± 1.74	8.37 ± 2.20	0.489
LYM, 10^9^/L	1.54 (1.22–1.96)	1.49 (1.17–2.02)	1.55 (1.27–1.86)	0.186
MONO, 10^9^/L	0.46 (0.35–0.56)	0.43 (0.32–0.56)	0.46 (00.40–0.56)	0.290
NEUT, 10^9^/L	3.78 (3.24–5.22)	3.57 (3.08–5.12)	4.05 (3.53–5.31)	0.067
RBC, 10^12^/L	4.44 ± 0.64	4.49 ± 0.60	4.38 ± 0.68	0.382
HsCRP, mg/L	2.26 (1.08–4.86)	1.89 (1.02–4.21)	3.08(1.19–6.47)	0.121
PLT, 10^9^/L	194.00 (157.00–217.50)	199.50 (158.50–216.50)	192.50 (154.50–223.25)	0.931

Abbreviations: SS, SYNTAX score. Other abbreviations are the same as in Table 1. *: *p* < 0.05.

**Table 3:** Assessment of independent predictors of CAD using univariate and multivariate logistic regression analysis.

**Variable**	**Univariate Model OR (95% CI)**	***p***	**Multivariate Model OR (95% CI)**	***p***
US-FLI	1.188 (1.188–1.365)	0.015 *	1.194 (1.008–1.414)	0.040 *
Gender	2.065 (1.179–3.616)	0.011	1.003 (0.431–2.335)	0.995
Age	1.051 (1.019–1.084)	0.002	1.054 (1.014–1.095)	0.008 *
Smoke	2.284 (1.229–4.247)	0.009	2.581 (1.016–6.552)	0.046 *
EF	0.962 (0.928–0.998)	0.037 *	1.000 (0.957–1.045)	0.993
T2DM	2.131 (1.160–1.160)	0.015 *	1.867 (0.885–3.936)	0.101
HBP	2.846 (1.579–5.131)	0.001	2.974 (1.463–6.046)	0.003 *
AST	1.013 (1.001–1.026)	0.032	1.011 (0.999–1.022)	0.075
HDL-C	0.262 (0.099–0.690)	0.007	0.370 (0.095–1.449)	0.154
ApoA1	0.207 (0.070–0.611)	0.004	1.371 (0.213–8.825)	0.740
ChE	0.876 (0.758–1.013)	0.073	0.868 (0.712–1.058)	0.161
NEUT	1.226 (1.038–1.448)	0.017	1.107 (0.899–1.364)	0.338
MONO	15.355 (2.389–98.703)	0.004	2.831 (0.277–28.914)	0.380

Abbreviations: CI, Confidence interval; US-FLI, ultrasonographic fatty liver indicator; EF, ejection fraction; T2DM, type 2 diabetes; HBP, hypertension; AST, aspartate aminotransferase; HDL-C, high-density lipoprotein cholestero; ApoA1, apolipoprotein A1; ChE, cholinesterase; NEUT, neutrophil; MONO, monocyte. *: *p* < 0.05.

**Table 4:** Collinearity diagnosis of all samples.

**Variable**	**VIF**
US-FLI	1.200
Gender	1.818
Age	1.365
Smoke	1.719
EF	1.197
T2DM	1.186
HBP	1.112
AST	1.184
HDL-C	1.720
ApoA1	2.070
ChE	1.362
NEUT	1.095
MONO	1.238

Abbreviations: VIF, variance inflation factor. The remaining abbreviations are the same as those in Table 3. When VIF < 10, there is no multicollinearity.

**Table 5:** Assessment of independent predictors of MS CAD using univariate and multivariate logistic regression analysis.

**Variable**	**Univariate Model OR (95% CI)**	***p***	**Multivariate Model OR (95% CI)**	***p***
US-FLI	1.225 (1.021–1.470)	0.029 *	1.262 (1.025–1.553)	0.028 *
Gender	2.031 (0.880–4.688)	0.097	1.868 (0.657–5.307)	0.241
EF	0.952 (0.909–0.998)	0.041 *	0.959 (0.909–1.011)	0.122
T2DM	4.974 (2.085–11.866)	<0.001 *	3.337 (1.226–9.085)	0.018 *
HBP	0.426 (0.170–1.065)	0.068	0.445 (0.147–1.349)	0.153
TBIL	0.951 (0.889–1.017)	0.139	0.954 (0.885–1.028)	0.218
ApoA1	0.310 (0.065–1.487)	0.143	1.223 (0.173–8.630)	0.840
Physical activity	0.490 (0.220–1.089)	0.080	0.471 (0.181–1.228)	0.124

Abbreviations: CI, confidence interval; US-FLI, ultrasonographic fatty liver indicator; EF, ejection fraction; TBIL, total bilirubin; T2DM, type 2 diabetes; HBP, hypertension; ApoA1, apolipoprotein A1. *: *p* < 0.05.

**Table 6:** Collinearity diagnosis of CAD samples.

**Variable**	**VIF**
US-FLI	1.047
Gender	1.217
EF	1.176
T2DM	1.274
HBP	1.128
TBIL	1.208
ApoA1	1.272
Physical activity	1.079

Abbreviations: VIF, variance inflation factor. CAD; coronary artery disease. The remaining abbreviations are the same as those in Table 5. When VIF < 10, there is no multicollinearity.

## Data Availability

The data from this study can be obtained through the first author.

## Supplementary Materials

The following supporting information can be downloaded at: https://www.mdpi.com/article/10.3390/diagnostics15101208/s1, Table S1: Intraclass correlation coefficient of US-FLI.

## Abbreviations

The following abbreviations are used in this manuscript:

ALP	Alkaline phosphatase
ALT	Alanine aminotransferase
ApoA1	Apolipoprotein A1
AST	Aspartate aminotransferase
BMI	Body mass index
CAD	coronary artery disease
ChE	cholinesterase
CI	Confidence interval
CVD	Cardiovascular disease
EAT	Epicardial adipose tissue
EF	Ejection fraction
FH	Familial heterozygous hypercholesterolemia
FLD	Fatty liver disease
GGT	Glutamyl transaminase
GLB	Globulin
HDL-C	High-density lipoprotein cholesterol
HGB	Hemoglobin
HsCRP	High-sensitivity C-reactive protein
ICA	Invasive coronary angiography
IR	Insulin resistance
LDL-C	Low-density lipoprotein cholesterol
Lp(a)	Lipoprotein(a)
LYM	Lymphocyte
MAFLD	Metabolic dysfunction-associated fatty liver disease
MetS	Metabolic syndrome
MONO	Monocyte
NAFLD	Nonalcoholic fatty liver disease
NEFA	Non-esterified fatty acid
NEUT	Neutrophil
OR	Odds ratio
PLT	Platelet
RBC	Red blood cell
RHR	Resting heart rate
ROC	Receiver operating characteristic
SS	SYNTAX score
T2DM	Type 2 diabetes mellitus
T-Cho	Total cholesterol
TG	Triglyceride
US-FLI	Ultrasonographic fatty liver indicator
VIF	Variance inflation factor
