# Supplementary material for: Positive Association Between Ultrasonographic Fatty Liver Indicator and the Severity of Coronary Artery Disease
Source: Diagnostics (Basel). 2025 May 10;15(10):1208. doi: 10.3390/diagnostics15101208 (PMC12110376; doi:10.3390/diagnostics15101208)
Supplement: Supplementary file 1 [file diagnostics-15-01208-s001.zip › diagnostics-3582353-supplementary.pdf]

Table S1. Intraclass correlation coefficient of US-FLI

	**Intraclass correlation coefficient**	**95% CI**	***P***
Total samples	0.948	(0.922-0.964)	<0.001

Abbreviations: US-FLI, ultrasonographic fatty liver indicator.
